# BioTCM-SE: A Semantic Search Engine for the Information Retrieval of Modern Biology and Traditional Chinese Medicine

**DOI:** 10.1155/2014/957231

**Published:** 2014-03-12

**Authors:** Xi Chen, Huajun Chen, Xuan Bi, Peiqin Gu, Jiaoyan Chen, Zhaohui Wu

**Affiliations:** College of Computer Science, Zhejiang University, Hangzhou 310027, China

## Abstract

Understanding the functional mechanisms of the complex biological system as a whole is drawing more and more attention in global health care management. Traditional Chinese Medicine (TCM), essentially different from Western Medicine (WM), is gaining increasing attention due to its emphasis on individual wellness and natural herbal medicine, which satisfies the goal of integrative medicine. However, with the explosive growth of biomedical data on the Web, biomedical researchers are now confronted with the problem of large-scale data analysis and data query. Besides that, biomedical data also has a wide coverage which usually comes from multiple heterogeneous data sources and has different taxonomies, making it hard to integrate and query the big biomedical data. Embedded with domain knowledge from different disciplines all regarding human biological systems, the heterogeneous data repositories are implicitly connected by human expert knowledge. Traditional search engines cannot provide accurate and comprehensive search results for the semantically associated knowledge since they only support keywords-based searches. In this paper, we present BioTCM-SE, a semantic search engine for the information retrieval of modern biology and TCM, which provides biologists with a comprehensive and accurate associated knowledge query platform to greatly facilitate the implicit knowledge discovery between WM and TCM.

## 1. Introduction

Recently, more and more biomedical researchers are focusing their attention on learning from complementary and alternative medicine (CAM) [1] in order to develop effective patient-targeted remedies, which is tagged as personalized medicine or integrative medicine. Traditional Chinese Medicine (TCM), as a widely known section of CAM, is characterized by the holistic understanding of the nature and the broad use of natural herbs. TCM is gaining increasing attention from Western healthcare practitioners because it is making favorable contributions to the development of novel drugs that are made of natural herbs. Thus, it will be quite useful to offer a TCM search platform for many modern biologists who want to explore the mystery of TCM. On the other hand, TCM has not been well adopted mainly due to the lack of scientific evidence, which can be potentially fulfilled by looking into the explicit and implicit connections to the well-recognized modern biology, for instance, the pharmacological basis of natural herb modulation. Many TCM physicians are eager to investigate and prove the effectiveness of the potential mechanisms of TCM from the perspective of modern biology. Therefore, it will be of great value to provide biologists with an association search engine for the information retrieval of modern biology and TCM to understand the functional mechanisms of the complex biological system as a whole.

However, with the fast development of modern biological data acquisition technologies, such as Next Generation Sequencing System (NGS) [2], huge volume datasets are generated in the life science domain. Up to now, Linked Life Data (LLD)—a data integration platform in the biomedical domain, has 10,192,505,364 statements (http://linkedlifedata.com/sources.html). Entrez Gene base maintains more 100 million gene records (http://www.ncbi.nlm.nih.gov/gene/) and UniProt Knowledge base (UniProtKB/Swiss-Prot) contains 34,535,400 sequence entries, comprising about 10 billion amino acids (ftp://ftp.uniprot.org/pub/databases/uniprot/relnotes.txt). Biomedical data also has a wide coverage across modern biology and TCM including proteins, pathways, diseases, targets, genes, Chinese Medical herbs, symptoms, and syndromes (http://en.wikipedia.org/wiki/Traditional_Chinese_medicine), which are scattered across numerous heterogeneous information systems, each with different terminologies, identifier schemas, and data formats. Moreover, different biomedical entities are implicitly connected by complex biological processes. Confronted with such large-scale, heterogeneous, interlinked, and complex-format biomedical data, it becomes quite difficult to construct a comprehensive and accurate knowledge query platform for associated TCMs and bioentities.

Traditional search engines cannot deal with the kind of associated knowledge search well since they are keyword based, which retrieve many inaccurate or incomplete documents for a user's keywords from lots of indexes. While this keyword-based search is fast and powerful for information retrieval, it is far from the vision of answering a user's questions by “understanding” the user's query and answers in the documents. For example, a biologist who is interested in* Artemisia annua* (a widely used herb to treat malaria) may ask these questions: “what is the mechanism of action of* Artemisia annua* from the point of modern biology?,” “which genes or proteins are related to* Artemisia annua*.” When a classical search engine is used to answer these questions by typing key words like “*Artemisia annua* modern biology,” “*Artemisia annua* gene,” or “*Artemisia annua* protein,” it returns many inaccurate and incomplete results since it only searches the keywords literally in text instead of semantically understanding the implicit relationships between these keywords. Therefore, the search engine does not know how to search answers in an appropriate way due to lack of enough biomedical background knowledge and the capability of semantic cognition.

Given the situation, it is difficult for biomedical researchers to utilize existing search engines to get available information of associated TCMs and bioentities from the large-scale, heterogeneous, and distributed biomedical data. There is an urgent need to present a novel search platform for the information retrieval of modern biomedical and TCM. Due to challenging big biomedical data searching efforts, a computational technique should satisfy the following three basic requirements:

- a standard and sharable conceptional model that is capable of capturing and modeling the comprehensive biomedical areas including modern biology and TCM;
- a data integration model to map and merge biomedical data into a unified warehouse across disparate data sources;
- a collection of search services to facilitate implicit knowledge discovery between TCM and modern biology.

The Semantic Web [3], proposed by Berners-Lee et al.—the inventor of the World Wide Web, has promising application potentials in life sciences and healthcare [4]. Firstly, the RDF (Resource Description Framework) [5], a graph-theoretic data model defined as a web technology standard, is ideally suitable for representing biomedical data model by subject-predicate-object triples. Each biomedical entity (e.g., gene, protein, disease, drug, Chinese herb, etc.) is mapped to a node and uniquely identified by a URI (Uniform Resource Identifier). The connections (e.g., part-of, treatment, and inhibit) between biomedical entities are captured by certain graph structures, such as a labeled directed link. The Semantic Web ontology languages, that is, RDFS (Resource Description Framework Schema) and OWL (Web Ontology Language), further enrich the biomedical model with formal vocabularies including classes, relationships, and properties [6]. The query language SPARQL is the standard for posing queries against repositories of semantic knowledge [7]. The semantic search promises to produce precise answers to user queries by taking advantage of the availability of explicit semantics of information in the context of semantic web. Enabling technologies such as ontology editors (e.g., Protege) [8], OWL reasoners (e.g., Pellet [9], Racer [10], and FaCT++ [11]), and triplestores with SPARQL endpoint (e.g., Virtuoso [12], AllegroGraph [13], and Sesame [14]) help make the Semantic Web vision a reality.

The Semantic Web technologies provide us with a good way to model the complicated and large-scale realms of biomedical knowledge network by defining a unified biomedical ontology based on domain knowledge. After creating the well-designed biomedical ontology, we have the ability to define some specific entities and declare common semantic relationships between existing entities. As a result, we are able to construct a big linked biomedical knowledge graph by interacting many heterogeneous biomedical data sources based on typed links defined in the unified biomedical ontology. Such links describe the certain relationships between biomedical entities, which are quite important for agents to provide expressive and accurate query capabilities over the data in the big biomedical knowledge graph.

In this paper, we present a novel semantic search engine* BioTCM-SE*, which aims at providing a comprehensive and accurate information retrieval system for large-scale modern biology data and TCM data. It is quite useful for biologists who focus on the association discovery between modern biology and TCM by offering easy and comprehensive search services. The implementation of* BioTCM-SE* prototype is mainly divided into three steps as follows. Firstly, semantic modeling method is used to create a unified biomedical ontology to model the complex biomedical domains by referencing several existing biomedical ontologies and manually constructing some new ontology descriptions based on corresponding domain knowledge. Secondly, we make full use of multiple text-processing techniques such as MapReduce [15] and Web Service to extract required large-scale biomedical instance entities into a unified linked biomedical knowledge graph, which acts as the data integration model of* BioTCM-SE*. Thirdly, we implement the semantic search services by extracting explicit semantic rules and executing customized SPARQL queries.

The major works in this paper are the following.

- We design a unified biomedical ontology to represent the complex and diverse biomedical domains including the modern biology and TCM by semantic modeling way. To the best of our knowledge, this is the first time that such comprehensive and complete biomedical ontology is presented.
- Based on the definition of the unified ontology, corresponding massive biomedical instance entities are collected into the backend knowledge repository of* BioTCM-SE* to form a big linked biomedical knowledge graph.
- We show the search functions of* BioTCM-SE* prototype by demonstrating several common use cases. These query results are quite useful for both TCM and modern biomedical researchers to understand the possible therapeutic mechanisms of TCMs from the perspective of modern biology, assist novel drug discovery by TCM remedies, and so on.
- We give the accuracy evaluation for multiple search results. The results show that* BioTCM-SE* achieves high precision.

The remaining of this paper is organized as follows.  Section 2 outlines related work, including existing semantic search engines and some biomedical data integration and search platforms. In Section 3, we give the overall architecture and detailed implementation of the* BioTCM-SE*.  Section 4 describes the results. Finally, we conclude and discuss the future work in Sections 5 and 6.

## 2. Related Work

### 2.1. Semantic Search Engines

There are a number of semantic search engines, such as Swoogle [16], Semantic Web Search Engine (SWSE) [17], WikiDB [18], Sindice [19], Watson [20], Falcons [21], and CORESE [22]. They include existing RDF repositories and crawl the internet for formal statements, for example, OWL files. A query retrieves a list of results with URIs. For SWSE and Falcon the result is enriched with a description and a filtering mechanism for result types. CORESE uses conceptual graphs for matching a query to its databases. WikiDB is slightly different from the others in that it extracts formal knowledge implicit in metatags of Wikipedia pages and converts it into RDF offering querying with SPARQL.

The above tools are intended to be general and as a result they generally lack corresponding biomedical knowledge background and cannot cover the biomedical domains well. Thus, they are not suitable for complex-associated biomedical knowledge query. Searching for example, for a TCM herb “*Ginkgo biloba*,” all the search engines only offer some basic information about the herb, but are not able to find other correlation information from external modern biomedical databases. It is mainly because an explicit semantic relationship between TCM and modern biology in their background semantic knowledge base does not exist.

### 2.2. Biomedical Data Integration and Search Platforms

Recently, several data integration and search platforms for the biomedical domain are presented, such as Linked Life Data (LLD) (http://linkedlifedata.com/), BioPortal [23], Bio2RDF [24], NCBI (http://www.ncbi.nlm.nih.gov/index.html), GeneCards [25]. LLD is a semantic data integration framework that enables access to multiple public biomedical databases. BioPortal is an open repository of biomedical ontologies that provides access via Web services and Web browsers to ontologies developed in OWL, RDF, and OBO format and Protege frames. Bio2RDF is a mashup system to help the process of bioinformatics knowledge integration. The NCBI is a system of interlinked biomedical databases created by the US National Library of Medicine, which provides a series of search services for biomedical data. GeneCards is a searchable, integrated, database of human genes that provides concise genomic related information, on all known and predicted human genes.

NCBI and GeneCards do not use semantic data warehouse to integrate biomedical databases, making it hard to represent and capture the complex semantic relationships between biomedical entities, which is the first basic requirement for BioTCM-SE as stated previously. LLD and BioPortal are both data-as-a-service platforms that provide access to many public biomedical ontologies. But they simply integrate these biomedical data, rather than constructing a standard and sharable conceptional model connecting modern biology to TCM. Furthermore, most WM experts are not familiar with TCM while TCM experts are also unfamiliar with WM. The factors make it very complicated and costly to manage and search in the comprehensive platform. So the creation of a large “merged” ontology is indispensable for our system. Bio2RDF builds a common ontology by parsing online fixed format HTML documents (from NCBI website or other websites). But the approach does not work when it comes to TCM because fixed format TCM HTML documents are not available online. And the approach also leads to some errors which might have some impact on the accuracy of our system.

Above systems also lack related TCM knowledge, leaving it impossible to construct a complete biomedical linked knowledge graph to provide efficient information retrieval of modern biology and TCM.

## 3. Architecture and Implementation of BioTCM-SE

**Figure 1:**
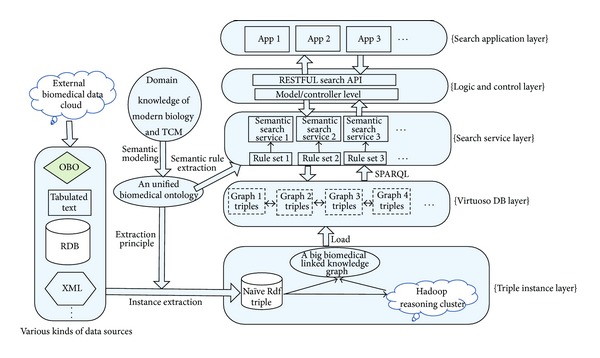
The overall architecture of BioTCM-SE.

Figure 1 shows the schematic description of* BioTCM-SE* architecture. The workflow of* BioTCM-SE* can be described as follows. The user submits a query by different search applications on the* BioTCM-SE* website to the server. The server preprocesses the query and invokes corresponding controllers to send a search request to the search service layer. Then corresponding search service will return the results to users by executing customized SPARQL query plan based on its semantic rule set.

The key functional modules of* BioTCM-SE* mainly consist of three parts: ontology modeling module, data integration module, and semantic search module. Ontology modeling module focuses on creating a unified biomedical ontology based on the domain knowledge of modern biology and TCM. Data integration module is used to collect all required data sources from external biomedical data cloud and transform these data with different formats (e.g., XML, Text, OBO, and RDB) to uniform triple format. Based on the conceptual model and data model, semantic search module is responsible for extracting semantic rule sets to accomplish search services by customized SPARQL query.

We first show the method used to build the ontology. Then we describe the process of data integration, including transform existing data into uniform RDF format, introduce the data storage strategy, and explain how we normalize URIs. At last, we present the concrete implementation of semantic search services.

### 3.1. Unified BioTCM Ontology

In this section, we introduce the function and design of the unified ontology, which we will refer to as Unified BioTCM Ontology in this paper.

#### 3.1.1. Ontology Function

The unified ontology model of* BioTCM-SE* is supposed to provide an explicit specification of the conceptualization of the abstract view of the integrated modern biology and TCM. For a given semantic knowledge base, it means that a conceptual language should be used to define the instances and the relationships to be represented.

Fundamentally, the Unified BioTCM Ontology should provide a common generalized terminological and assertional base for mapping from multiple sources to a unified mapping schema. More importantly, it also needs to portray the relations among pairs of TCM or modern biological entities.

Unified BioTCM Ontology is a crucial component of* BioTCM-SE*, playing a fundamental role in integrating multiple ontologies and extracting search rules; that is, (1) it is a unique ontology, which captures the synonym concepts and crucial properties that help build the theoretical model of integrated knowledge of modern biology science and TCM; (2) it provides a communication boundary for those who are interested in but do not know much about the consensus and differences among multiple disciplines of knowledge, especially; (3) it defines the explicit semantic relations between different biomedical entities, which enables more effective and accurate search services for cross-domain-associated knowledge query.

#### 3.1.2. Ontology Design

The Unified BioTCM Ontology is mainly a terminology box which consists of class hierarchies and class restrictions defined with object properties. Since the main goal of Unified BioTCM Ontology is to unify the distributed ontologies on the web into an integrated linked knowledge graph, the unified ontology also provides a unique URI identity for every biomedical resources (e.g., entities, relationships, classes, etc.). The normalization of the URIs will be discussed in detail in the next section.

To consider the relation between expressivity and complexity in ontologies is very important when we construct and manage the Unified BioTCM Ontology. There is a positive correlation between expressivity and complexity of ontology. If we reduce the expressivity of the ontology, we might affect the type of services it can offer. Otherwise, if we construct a very complex ontology, it will be very difficult to manage the ontology. An ideal balance between expressivity and complexity should provide us with a simple and efficient way to represent and manage the big knowledge graph without affecting the required services. In practice, the balance largely depends on the application scenarios.

Specifically speaking, as the main goal of the BioTCM-SE is to discover the implicit association knowledge between TCM and modern biology by joining multiple intermediate terms, one of the most important works we have to do is to capture the transitive relations in our unified ontology. RDF and RDFS are not able to express the semantic information due to limited expressivity, while OWL uses the vocabulary “TransitiveProperty” to represent the transitive properties to form the property chains (rule sets). The search rule sets provide evidence to transform corresponding customized SPARQL queries for search service module. On the other hand, under the premise in expressivity, we want to reduce the complexity of the ontology as much as possible. OWL provides three increasingly expressive sublanguages designed for use by specific communities of implementers and users. We chose the simplest OWL Lite language to construct the ontology. If we want to offer more services such as reasoning or computing closure, we need to choose more complex OWL language like OWL DL and OWL Full. To design the Unified BioTCM Ontology, we used the Protege open source framework and its OWL editor Protege-OWL.

**Figure 2:**
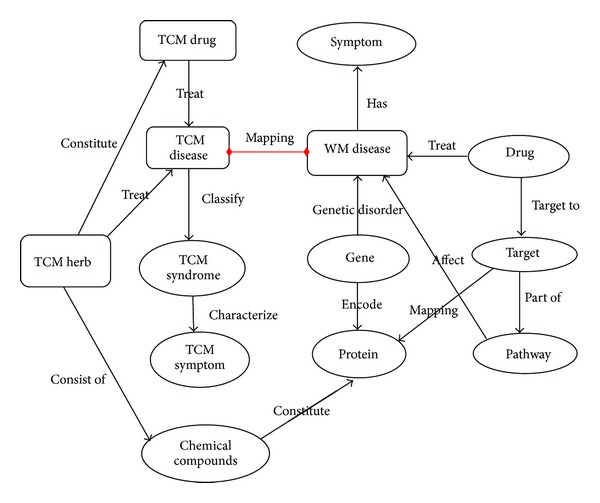
The basic associations that are concerned and observed between Traditional Chinese Medicine and modern biology.

The design of Unified BioTCM Ontology was inspired by already existing ontologies and domain knowledge of multiple disciplines. In our* BioTCM-SE* platform, the unified ontology is mainly composed of two top-level conceptual classes: biotcm:unified model and biotcm:data sets. The idea is simple: biotcm:unified model represents the core theoretical foundation of how the modern biology knowledge and basic theories and clinical wisdom of traditional Chinese medicine should be connected and interlinked, the class biotcm:data sets are used to keep track of the knowledge sources that are being integrated, and their concerned concepts and attributes (e.g., Gene Ontology, Uniprot, etc.).

biotcm:unified model conceptualizes the association network among concerned entities, for example, disease, drug, gene, herbs, at a high level of integrity.  Figure 2 gives a brief description of the association network model. Under the category of biotcm:unified model, there are many concepts that we consider of significant importance during data integration: Disease, Drug, Gene, Protein, Pathway, Symptom, Syndrome, Target, TCM Herb, TCM Symptom, TCM Syndrome, and so on. Mainly, specific disorders of certain genes can affect the encoding proteins, which cause diseases to appear. Drugs are used to treat diseases by interacting with the influential proteins through possible targets and involved pathways. The herbs are the constituents made up of drugs. The major link between modern biology and Chinese medicine lies in the fact that some Western diseases are similar to some TCM diseases, and it has been found that certain genes are responsible for some TCM diseases and that certain remedies (e.g., herbs) might cure the genetic disease by possible biological targets.

biotcm:data sets are created in two ways. For the data source whose OWL schema is available online (most modern biology data), we directly extract some useful definitions from their OWL schemas. For example, we can get the descriptions of gene products from Gene Ontology (http://www.geneontology.org/GO.downloads.ontology.shtml) and further transform the information into the biotcm:data. For other data sources which lack ontology definitions (most TCM data), we manually add key concepts and attributes with the help of related domain experts into biotcm:data. For example, we refer to three TCM-related data sources:* BioTCM:TCMGeneDIT* [26],* BioTCM:TCMLS* [27], and* BioTCM:FivePhases* [28]. Then TCM experts help us extract important TCM concepts and properties from the three data sets as a part of biotcm:data.

### 3.2. Biomedical Data Integration

After we have designed a well-defined comprehensive biomedical ontology, the ontology tells us which data needs to be collected and how their schemas should be. With the information, data integration module is able to integrate required data sources to form a big linked biomedical knowledge graph. The left part of Figure 1 gives a system overview of our semantic approach to data integration. It entails the following steps.

- Download the contents (e.g., txt, xml, obo, RDB, ASN.1, and KGML) from various kinds of heterogeneous data sources and convert the downloaded data into our RDF format based corresponding ontology schema.
- Normalize URIs to allow proper connection of triples and facilitate query.
- Load the RDF-formatted data files into the semantic database for data storage, management, and retrieval. Once the big linked biomedical knowledge graph is formed, (web-enabled) search applications can be written to allow users to access accurate query results.

#### 3.2.1. Data Extraction and Conversion

The scientific focus of* BioTCM-SE* search engine is to provide a comprehensive search service across modern biology and TCM knowledge for biomedical researchers. In an attempt to force the design to be general, we must provide sufficient data sets that will support our focus.

There are numerous bioinformatics databases available for modern biology. Although RDF was proposed as a standard format for the web, these databases are still available in various formats. We utilize many different ways to extract these data sources. For example, for some small text data sources, we use simple text mining method to extract required instance triples. For some large-scale text data sources, such as Uniprot, we have to utilize some big data computing framework to finish the task. We use MapReduce parallel computing framework to extract required information into semantic knowledge graph. For the data from relational databases, we use RDB2RDF tools such as D2R [29] to enable this data into standard RDF data. We also acquire some online data by Web Service, such as the NCBI efetch service (http://www.ncbi.nlm.nih.gov/books/NBK25501/). Then we transform these data to RDF format. As a result, our knowledge base almost covers all the domains of modern biology, including gene, protein, pathway, drug, disease, target, and clinical trial.

On the other hand, TCMGeneDIT, the most popular TCM information database, which collects information from public databases, including TCM herb database HULU, TCM-ID, NCBI Entrez Gene, and medical subject headings vocabulary (Mesh), forms a unique mixed database that offers diverse association information related to TCM and modern biology. It builds an ideal bridge to link TCM knowledge with the knowledge of modern biology. As for TCM knowledge, we mainly collect data from the Traditional Chinese Medicine Language System—the largest and most authoritative Traditional Chinese Medicine ontology all over the world by now. It is a comprehensive language system about TCM, curated by domain experts in China based on a grid infrastructure, covering large amount of facts collected from ancient corpus, clinical trials. Besides that, we also add other TCM data sources into the knowledge graph, such as FivePhases ontology data.

In addition to above data sets, some implicit knowledge derived by distributed reasoning over naive triples is also integrated into the big linked graph.

#### 3.2.2. URI Normalization

For our semantic search engine, normalized representation of URI is necessary to ensure a semantic connection between triples. A central tenet of the Semantic Web is that entities (known as “resources” in web parlance) are identified or named by URIs. The identity of entity is essential for knowledge sharing and searching. When biologists use the same names for the same biomedical entity, they can more easily share, analyze, and search knowledge. Although we have transformed all data into semantic triples, every entity in the triple, expressed as URIs, still needs to be normalized to allow proper connection of triples. For example, a PubMed resource with an identifier 12728276 can be referenced with PMID:12728276, PubMed:12728276, or PubMed:12728276. Even in existing well-formed RDF documents there is a problem with URIs. For example the GO term of* transcription elongation from mitochondrial promoter* (GO:0006392) is referenced by different URIs used by existing RDF data providers: Uniprot, OBO, and BioPathways Consortium:

 http://www.geneontology.org/go#GO:0006392
 
http://www.ebi.ac.uk/QuickGO/GTerm?id=GO:0006392
 
*urn:lsid:geneontology.org.lsid.biopathways.org:go:0006392*

These URIs are all supposed to represent the same entity: the definition of* transcription elongation from mitochondrial promoter biological process* according to Gene Ontology. However, when we load corresponding data sources into the knowledge graph, no links would be created among them because their URIs are different even if they are mapped to the same concept. To solve the problem, the* BioTCM-SE* normalizes the URI pattern for all URIs regardless of the provider. Specifically, we normalize the URIs of the resources to fit to the BioTCM ontology syntax; that is, every resource node will be allocated with a unique* biotcm* identity. For example, a gene originally defined in the Gene Ontology was tagged with a GO id #goid but should be assigned with a Unified BioTCM Ontology identifier “http://www.biotcm.org/go/#goid.” The normalization of the URIs will be obtained through the assertion of an object property biotcm:url. So our system guarantees that the semantic connections are built automatically around the same entity. Based on the rule, the URI for the mentioned GO: 0006392 is expressed as http://www.biotcm.org/go/#0006392. As the ontology has not been published as linked data, the URIs can not be accessible for the moment.

When a new data source is loaded, we apply the URI syntax to create a unique identity for every entity in the data source. At the same time, to keep track of official URI, we add an owl:sameAs predicate linking to the normalized entity URI. The use of normalized identifiers has shaped a big linked biomedical knowledge graph in the true sense, which provides a unified data model for further semantic search.

#### 3.2.3. Data Storage

After creating the linked biomedical knowledge graph, we need to choose appropriate semantic database to load this graph. Openlink's Virtuoso, an innovative and distributed multimodel server, offers an ideal linked data management platform. Each data source in the linked knowledge graph is regarded as a bundle. The largest bundle of 62 M triples contains the relations between protein and gene product. Each converted data source in the linked knowledge graph is put in a distinct but interconnected named graph. These graphs are similar to the tables of relational database. The Virtuoso Database layer provides a semantic query interface to access these triples in the named graphs for upper search service layer via SPARQL.  Table 1 lists some major graphs currently deployed in the* BioTCM-SE*.

### 3.3. Semantic Search Module

The semantic search module provides a list of search services which allow users to retrieve more precise answers by taking advantage of the availability of explicit semantics of information in the context of the Unified BioTCM Ontology and big linked graph. In the* search service layer*, every semantic search service corresponds to a rule set which refers to the semantic rules defined in the Unified BioTCM Ontology. Subsequently, these rule sets call customized SPARQL queries to tell the search engine how to fetch the results from the large-scale graph triples.

We consider this search service request: search possible Traditional Chinese herbs related to gene Interleukin-6 (IL6). Simple querying Google or other search engines with a phrase such as “IL6 and herb” or “IL6 and TCM” yields many incomplete and uncorrelated results. In contrast, our semantic search engine can get more complete and more scientific results based on a semantic search chain that can capture explicit semantic association between gene and Traditional Chinese herb.

**Figure 3:**
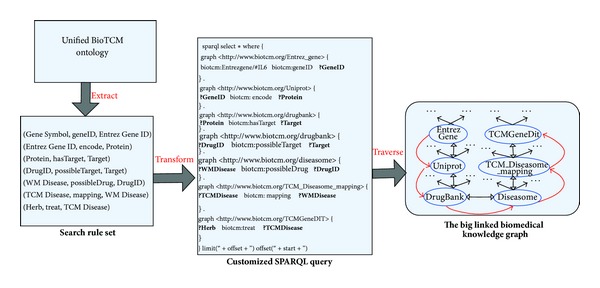
The workflow for searching possible herbs related to gene Interleukin 6 (IL6).

Specifically, when users submit above search request,* search service layer* first needs to extract semantic rules from the Unified BioTCM-SE Ontology. According to the implicit semantic association between herb and gene that is defined in the Unified BioTCM-SE Ontology, corresponding rule set is determined. Then the rule set transforms into a customized SPARQL query which traverses six graphs within the big linked knowledge graph (Figure 3):* Entrez Gene* (defining the term IL6 and its Gene ID),* Uniprot* (mapping between protein and Gene ID),* Drugbank* (including drug, target, and protein information),* Diseasome* (connecting WM disease and drug),* TCM-Diseasome *(mapping between WM disease and TCM disease), and* TCMGeneDit *(including herb and TCM disease). At last, the search engine fetches related herbs and returns the results to users. To have a comprehensive understanding of the interaction process between gene and herb,* BioTCM-SE* also returns many intermediate results such as related proteins, diseases, and targets. If users only want the information about herbs, they only need to click button “TCM Name” to filter other intermediates.

## 4. Results

The* BioTCM-SE* project is still under development, but many functionalities have been made available to the public. It offers a web-based application that users can browse and query comprehensive biomedical knowledge through a single access point (http://www.biotcm.org/biotcm-se/indexpage/index.html (112.124.0.23/biotcm-se)). The data collected from multiple sources is deployed on Ali Cloud storage service platform. As for GUI representation, we use the Twitter Bootstrap javascript framework to build the application, and we use Cytoscape visualization package to paint the knowledge graphs.

The goal of* BioTCM-SE* is to provide biologists with a comprehensive and accurate association query platform for the information retrieval of modern biology and TCM. The precondition to achieve the goal is to construct a biomedical data integration platform across disparate data sources. Therefore, we will show the results from three aspects. Firstly, we give the detailed description of the large-scale linked biomedical knowledge graph which serves as the background semantic knowledge base of* BioTCM-SE*. Then, we present the search services provided by* BioTCM-SE* with several concrete use cases. At last, we give the accuracy evaluation based on the results from the use case studies.

### 4.1. The Big Linked Biomedical Knowledge Graph

**Figure 4:**
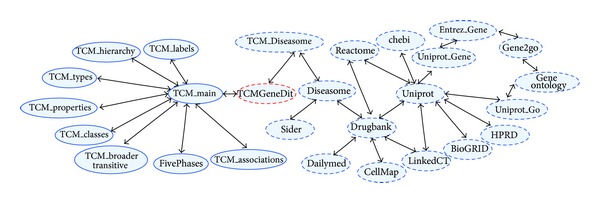
The big linked biomedical knowledge graph of BioTCM-SE.

Presently, our linked biomedical knowledge graph consists of more than 25 typical bioinformatics data sources which have a wide coverage of diverse biomedical domains. These data sources are now available from the biotcm.org server in a normalized RDF format based on previously designed unified ontology. It is a massive knowledge base of millions of RDF triples. To sum up, there are currently 120 million triples occupying more than 15 gigabytes in the knowledge base.  Figure 4 shows the big linked biomedical knowledge graph.

### 4.2. Use Cases

The* BioTCM-SE* provides a list of search services for comprehensive biomedical entities including TCM herb, gene, gene product, disease, drug, target, and protein. The search engine mainly focuses on the association knowledge search of TCM and modern biology. To be specific, search services offered by* BioTCM-SE* mainly fall into three classes:* Bio for TCM*,* TCM for bio*, and* comprehensive terminology query*. The first kind of search service lists out the related modern biomedical resources about queried TCM herbs, including possible affecting diseases, contributing drugs, associated targets and proteins, related genes, and gene products (cellular components, biological processes, and molecular). Similarly, the second kind of search service can return the corresponding TCM information for certain drugs, genes, proteins, GO ids, and diseases. The last kind of search service retrieves all associated information about some biomedical entity. Moreover, our search engine offers straightforward graph visualization and zoomable network for query results. The visualization function provides a useful filtering capability which allows users to select interesting entity to further browse and query.

We will demonstrate these functions by the following practical case studies.

#### 4.2.1. *Bio for TCM* Case Study

We present* Bio for TCM* case study performed on the search engine by discovering the associations from TCM herbs to genes through intermediate diseases. We selected several popular TCM herbs which have been studied a lot: Artemisia annua, Aconitum carmichaelii, Ginkgo biloba, and Salvia miltiorrhiza. According to our association rule set, the association information includes diseases, proteins, genes, gene products, and corresponding number of associated chains.

As listed in Table 2, for example, Artemisia annua is actually a natural plant whose extract artemisinin is the standard treatment worldwide for* P. falciparum malaria*. In our system, we found 58 related statements, involving proteins like Hemoglobin subunit alpha, and genes like MMP9, COL13A1. More discoveries can be found in the results, for instance, Artemisia annua has been found to have connections to breast neoplasm, colonic neoplasm, leukemia, lung neoplasm, malaria cerebral, malaria falciparum, malaria vivax, melanoma, and ovarian neoplasm. Another famous Chinese herb Ginkgo biloba is known to cause moderate cognition improvement for Alzheimer's disease patients with dementia symptom, while in our system, it associates with 161 genes.

These results might help researchers have a deeper understanding of TCM herbs from the perspective of modern biology so as to promote the modernization of TCM.

#### 4.2.2. *TCM for Bio* Case Study

Similarly,* TCM for Bio* case study is presented by finding the implicit TCM herbs related to certain genes through intermediate diseases. We also selected several major reported genes: IL6, TNF, IFNG, and MMP9. The search results are listed in Table 3. Take gene Interleukin-6 (IL6) for example. IL6 is a pleiotropic cytokine with obviously tumor-promoting and tumor-inhibitory effects, which has been widely regarded to be closely correlated with neoplastic diseases such as Prostatic Neoplasm and Breast Neoplasm [30, 31]. Our search results reveal that there are 46 herbs associated with gene IL6 through disease Breast Neoplasm including* Ganoderma lucidum*,* Salvia miltiorrhiza*, and* Hypericum perforatum*. On the other hand, according to analysis of the chemical components in these herbs, most of these herbs contain anticancer compounds. The compounds can cause breast cancer cells to round up and die, inhibit tumor-induced blood supply development, and prevent tumor growth [32–34]. Other results show that gene tumor necrosis factor (TNF) has connections to 16 TCM herbs with disease Stomach Neoplasm and that gene Interferon gamma (IFNG) associates with 5 TCM herbs through disease Vascular Disease.

These associations suggest the possible therapeutic mechanisms involved by herbs, disease, genes, and herb ingredients. The results also might be useful for researchers to assist the development of novel integrative drug.

#### 4.2.3. Comprehensive Terminology Search

If one wants to search a specific term or id from all the deployed sources,* Term Search* is a nice tool to give a knowledge graph view of the term. At present, our search engine supports disease term search, Gene Ontology term search, TCM term search, drug term search, protein term search, and gene term search.

### 4.3. Accuracy Evaluation

The accuracy evaluation is based on the association results from Tables 2 and 3. As there is not a gold standard for determining the correct mapping space between TCM and WM, the recall and F1-measure evaluation cannot be performed. We manually evaluate the results with the help of many biomedical experts and existing online medical materials. If there are too many association results (samples) about queried gene or herb, we will randomly select 50 samples from the set of results to evaluate. We use precision measurement to estimate the accuracy. Three annotators with graduate degrees in biomedical and TCM domains independently examined whether each pair was correctly extracted by our system. Only the pairs agreed upon by all three curators were counted as true positives (TP). Precision is defined according to formula (1), where TP and FP are the numbers of true positives and false positives, respectively. All the search results are available online (https://github.com/hualichenxi/BioTCM-SE). Users also can get the same results by corresponding search service in the* BioTCM-SE*. Consider

(1) $$\begin{array}{c} \text{Precision}=\frac{\text{TP}}{\text{TP}+\text{FP}}. \end{array}$$

Generally speaking, Table 4 shows that our results achieve high precision. But for the entities that have more 50 associated genes or herbs (such as Lines 1, 3, 5, and 8 in Table 4), the precision is relatively low. On the contrary, the precision is very high for other entities having few mappings (Lines 2, 4, 12, 13, 14, and 16 in Table 4). It is mainly because many mappings between TCM and WM are still unproven by professional biochemistry experiments. So more samples are more likely to contain more FP. It also means that calculated precision is underestimated because we may mistake some TP for FP in the manual evaluation. In fact, those undiscovered relationships between TCM and WM might be more useful to inspire biologists in the development of novel drugs and the modernization of TCM.

To sum up, our* BioTCM-SE* system can provide biologists with a comprehensive, reliable, and accurate associated knowledge query platform for the information retrieval of modern biology and TCM.

## 5. Discussion and Conclusion

Faced with the challenges for the analysis and mining of large-scale heterogeneous biomedical data, it will show great value to provide a big biomedical data management and query platform. In this paper, we have presented* BioTCM-SE*, a semantic search engine for the information retrieval of modern biology and TCM. The primary goal of this search engine is to provide biologists with a comprehensive and accurate query platform for associating TCMs and bioentities. Specifically, we first create a unified biomedical ontology that models the conceptual hierarchies and existential property restrictions that best describe the connections between modern biology and Traditional Chinese Medicine. It models the complex and diverse biomedical domains across the modern biology and TCM. Then, based on the definition of the unified biomedical ontology, required massive biomedical data is collected and integrated into the backend knowledge repository of* BioTCM-SE* to form a big linked biomedical knowledge graph. Data sources mainly come from open data repositories such as Gene Ontology and Uniprot, that describe the terminologies and vocabularies of biological disciplines, and well-formed ontologies created for the basic theories and therapeutic wisdom of traditional Chinese medicine (e.g., TCMLS, TCMGeneDIT, FivePhases ontology). Subsequently, we describe the semantic search module of* BioTCM-SE* which allows users to access the big linked biomedical knowledge graph by several search service interfaces. At last, results are presented by demonstrating the large-scale linked biomedical knowledge graph, several common association search case studies, and accuracy evaluation.

## 6. Future Work

For future work, firstly, due to the lack of sufficient biomedical background knowledge, we do not make full use of integrated knowledge in the* BioTCM-SE*. So we will consult more biomedical experts to help perfect the system by offering more accurate and more scientific search services. Besides, we will invite more bioinformaticians and biomedical researchers to complement the Unified BioTCM Ontology and work on collecting data and clarifying the biological process that TCMs could influence or participate, and thus to support more comprehensive association search services to fill the knowledge gap and enhance comprehensive life science. At last, since most of the biomedical data still exists in scientific literatures (such as PubMed), we plan to add text search and text mining services for the BioTCM-SE based on existing knowledge graph and literature databases.

## Figures and Tables

**Table 1:** Major data sets that have been integrated in BioTCM-SE.

Graph	Number of triples	Size (MB)
http://www.biotcm.org/TCM_hierarchy	177,960	7.4
http://www.biotcm.org/TCM_classes	790	0.03
http://www.biotcm.org/TCM_broaderTransitive	975,478	21.9
http://www.biotcm.org/TCM_types	119,409	5.1
http://www.biotcm.org/TCM_labels	501,081	21.3
http://www.biotcm.org/TCM_associations	294,219	8.0
http://www.biotcm.org/TCM_properties	720	0.03
http://www.biotcm.org/TCM_main	902,413	41.3
http://www.biotcm.org/TCMGeneDit_TCMLS_mapping	461	0.02
http://www.biotcm.org/TCMGeneDit	111,939	21.9
http://www.biotcm.org/TCM_Diseasome_mapping	2,699	0.2
http://www.biotcm.org/diseasome	72,445	15
http://www.biotcm.org/drugbank	517,023	98.3
http://www.biotcm.org/sider	4,054,800	18.6
http://www.biotcm.org/cellmap	149,175	20
http://www.biotcm.org/LinkedCT	25,762,568	1,228.8
http://www.biotcm.org/dailymed	162,972	114.5
http://www.biotcm.org/Uniprot_GO_mapping	62,209,291	8,499.2
http://www.biotcm.org/Uniprot_gene_mapping	9,444,045	1,331.2
http://www.biotcm.org/Entrez_Gene	7,171,247	1228.8
http://www.biotcm.org/go	1,931,018	237.6
http://www.biotcm.org/gene2go	1,351,005	207.8
http://www.biotcm.org/BioGrid	1,961,200	1,738.8
http://www.biotcm.org/HPRD	2,699	271.8
http://www.biotcm.org/chebi	323,212	38.3
http://www.biotcm.org/Reactome	1,082,499	110.7

**Table 2:** Examples of TCM herb-related diseases and bioentities: proteins, genes, and gene products.

Herb	Diseases	Number	Proteins	Genes	Gene products
*Artemisia annua *	Malaria	58	Hemoglobin subunit alpha, Lymphotoxin-beta	COL13A1, MMP9	Apical constriction, apyrase activity, pseudohyphal growth

*Artemisia annua *	Breast neoplasm	9	Sex hormone-binding globulin, Prolactin receptor	SHBG, PRLR	Protein complex assembly multichaperone pathway

*Aconitum carmichaelii *	Hypertension	179	Mineralocorticoid receptor, endothelial Nitric oxide synthase	NR3C2, NOS3	Ethanolamine-phosphate cytidylyltransferase activity, urate oxidase activity

*Aconitum carmichaelii *	Arthritis	13	Endothelial Nitric oxide synthase, Uridine-cytidine kinase 2	NOS3, UCK2	Phospholipid biosynthetic process, ventral midline determination

*Ginkgo biloba *	Alzheimer's disease	161	Urokinase-type plasminogen activator, Tumor necrosis factor	PLAU, TNF	Neurotransmitter: sodium symporter activity, pseudohyphal growth

*Ginkgo biloba *	Atherosclerosis	24	Prostaglandin G/H synthase 1, Cytosolic phospholipase A2	VEGFA, PLA2G4A	Peripheral nervous system development, high-density lipoprotein

*Salvia miltiorrhiza *	Asthma	92	Phenylalanine-4-hydroxylase, Vascular endothelial growth factor A	PAH, VEGFA	Protein kinase C activity, type II intermediate filament associated protein

*Salvia miltiorrhiza *	Parkinson's Disease	104	Mitochondrial Glutamate dehydrogenase 1, Estrogen receptor	GLUD1, ESR1	Antigen processing and presentation following pinocytosis, tRNA wobble guanine modification

**Table 3:** Examples of gene-related drugs, diseases, and TCM herbs.

Gene	Protein	Drugs	Diseases	Number	Herbs
*IL6 *	Interleukin-6	Bicalutamide	Breast Neoplasm	46	*Ganoderma lucidum, Salvia miltiorrhiza, Hypericum perforatum *

*IL6 *	Interleukin-6	Bicalutamide	Prostatic Neoplasm	22	*Ganoderma lucidum, Humulus lupulus, Belamcanda Chinensis *

*TNF *	Tumor necrosis factor	Adalimumab	Asthma	26	*Ginkgo biloba, Cannabis sativa *
					*Salvia miltiorrhiza *

*TNF *	Tumor necrosis factor	Procaterol	Stomach Neoplasm	16	*Panax ginseng, Curcuma longa Glycyrrhiza uralensis *

*IFNG *	Interferon gamma	Simvastatin	Vascular Disease	5	*Salvia miltiorrhiza, Allium sativum Ginkgo biloba *

*IFNG *	Interferon gamma	Glucosamine	Tuberculosis	11	*Sus Scrofa, Panthera leo Cervus elaphus *

*MMP9 *	Matrix metalloproteinase-9	Simvastatin	Alzheimer Disease	23	*Ginkgo biloba, Panax ginseng Polygonum multiflorum *

*MMP9 *	Matrix metalloproteinase-9	Minocycline	Diabetic Retinopathy	2	*Atractylodes lancea, Ginkgo biloba *

**Table 4:** Accuracy evaluation of the associations between TCM herbs and genes.

Herb/gene	Disease	Sample (number of related genes/herbs)	TP	Precision
*Artemisia annua *	Malaria	50	40	80%
*Artemisia annua *	Breast Neoplasm	9	9	100%
*Aconitum carmichaelii *	Hypertension	50	38	76%
*Aconitum carmichaelii *	Arthritis	13	10	76.92%
*Ginkgo biloba *	Alzheimer disease	50	43	86%
*Ginkgo biloba *	Atherosclerosis	24	21	87.5%
*Salvia miltiorrhiza *	Asthma	50	43	86%
*Salvia miltiorrhiza *	Parkinson Disease	50	37	74%
*IL6 *	Breast Neoplasm	46	39	84.78%
*IL6 *	Prostatic Neoplasm	22	19	86.36%
*TNF *	Asthma	26	23	88.46%
*TNF *	Stomach Neoplasm	16	15	93.75%
*IFNG *	Vascular Disease	5	5	100%
*IFNG *	Tuberculosis	11	11	100%
*MMP9 *	Alzheimer Disease	23	18	78.26%
*MMP9 *	Diabetic Retinopathy	2	2	100%

*Sum up *		447	373	83.45%
